# Comparative efficacy of intratympanic gentamicin and intratympanic corticosteroid in the treatment of Meniere’s disease: a systematic review and meta-analysis

**DOI:** 10.3389/fneur.2024.1471010

**Published:** 2024-09-12

**Authors:** Xuanmei Wu, Jiacheng Shui, Chengyuan Liu, Xinyue Wu, Ying Yu, Hanyu Wang, Cong Yan

**Affiliations:** ^1^School of Life Sciences, Beijing University of Chinese Medicine, Beijing, China; ^2^Eye Hospital China Academy of Chinese Medical Sciences, Beijing, China

**Keywords:** intratympanic gentamicin, intratympanic corticosteroid, Meniere’s disease, meta-analysis, systematic review, efficacy

## Abstract

**Purpose:**

We aimed to evaluate the efficacy of gentamicin compared to corticosteroids for the treatment of Meniere’s disease.

**Methods:**

An extensive search was conducted in PubMed, Embase, and Web of Science until May 2024. For continuous outcomes, pooled effect estimates were determined by calculating the weighted mean difference (WMD), while for binary outcomes, the risk ratio (RR) was used, each accompanied by their respective 95% confidence intervals (CIs). Heterogeneity among the studies was assessed using Cochran’s *I*^2^ and *Q* statistics.

**Results:**

A total of 12 studies were selected, involving 694 patients. Our analysis found that the gentamicin group demonstrates superior vertigo control rates compared to the corticosteroid group (RR: 1.36, 95% CI: 1.13 to 1.65, *p* < 0.001). In subgroup analysis, the gentamicin group showed a higher vertigo control rates at 6 months compared to the corticosteroid group (RR: 1.69, 95% CI: 1.28 to 2.24, *p* < 0.001); however, there was no statistically significant difference between the two groups at 12 months (RR: 1.48, 95% CI: 0.88 to 2.49, *p* = 0.14). Regarding changes in pure tone average, the corticosteroid group was superior to the gentamicin group (WMD: 4.41, 95% CI: 3.31 to 5.52, *p* < 0.001).

**Conclusion:**

Our study suggests that the intratympanic gentamicin group achieves higher vertigo control rates, whereas the corticosteroid group demonstrates better improvement in pure tone averages. However, the high heterogeneity in vertigo control rates warrants caution. Larger sample-sized randomized controlled trials are needed to further validate these findings.

## Introduction

Meniere’s disease is a chronic disorder of the inner ear characterized by episodes of vertigo, fluctuating hearing loss, tinnitus, and a sensation of fullness in the ear (1). This debilitating condition affects a significant portion of the population (2). The global prevalence of Meniere’s disease ranges from 50 to 200 cases per 100,000 people (3). The etiology of Meniere’s disease remains poorly understood, though it is believed to involve abnormal fluid dynamics within the inner ear (4). Early treatment is crucial in managing symptoms, preventing the progression of hearing loss, and improving patients’ quality of life (2).

Current pharmacological treatments for Meniere’s disease primarily include diuretics and betahistine to manage vertigo symptoms (5). Diuretics aim to reduce fluid buildup in the inner ear, while betahistine is believed to improve blood flow within the ear and reduce pressure (6). In cases where pharmacological therapy proves ineffective, surgical interventions such as endolymphatic sac decompression, vestibular nerve section, and labyrinthectomy may be considered (7, 8). However, these approaches have limitations, including the potential for significant complications and variable long-term efficacy (9). For instance, while endolymphatic sac decompression is less invasive, its benefits are often temporary (7). Vestibular nerve section and labyrinthectomy, though more definitive, carry risks of permanent hearing loss and other complications (10). Furthermore, the invasiveness of surgical procedures often deters patients from opting for these treatments, highlighting the need for effective non-surgical alternatives.

Intratympanic corticosteroids and intratympanic gentamicin have emerged as promising alternatives for the management of Meniere’s disease (11, 12). Intratympanic corticosteroids, such as dexamethasone, are believed to reduce inflammation and autoimmune responses within the inner ear, providing symptomatic relief with a favorable safety profile (13). Intratympanic gentamicin, an aminoglycoside antibiotic, works by selectively ablating vestibular hair cells, thereby reducing vertigo episodes (14). Despite their potential benefits, the efficacy of these intratympanic treatments remains a topic of debate within the medical community. The choice between these treatments involves a careful consideration of the potential for vertigo control versus the risk of hearing loss (15, 16). Previous meta-analyses have reported varying outcomes, leading to ongoing controversy regarding which treatment offers superior efficacy and safety (17, 18).

The purpose of this meta-analysis is to evaluate and compare the efficacy of intratympanic gentamicin and intratympanic corticosteroid in the treatment of Meniere’s disease.

## Materials and methods

The meta-analysis strictly adhered to the 2020 guidelines outlined by the Preferred Reporting Items for Systematic Reviews and Meta-Analyses (PRISMA) (19). Furthermore, the protocol for this meta-analysis was registered with PROSPERO, identified by CRD42024557594.

### Search strategy

A comprehensive search was conducted across multiple databases, including PubMed, Embase, and Web of Science, for relevant publications up to May 2024. This search employed specific keyword terms: (“Meniere’s Disease”) AND (“Gentamicin” OR “Gentamycin”) AND (“Steroids” OR “Glucocorticoids”). The search terms used in this study are detailed in Supplementary Table S1. Additionally, the reference lists of the selected articles were manually reviewed to identify further relevant research.

### Inclusion and exclusion criteria

The following criteria were applied: Population (P): Patients with Meniere’s disease; Intervention (I): Intratympanic Gentamicin; Control (C): Intratympanic Corticosteroid; Outcomes (O): (1) vertigo control rates; (2) changes in pure tone average; Study design (S): Randomized controlled trials (RCTs), retrospective studies, and prospective studies.

The exclusion criteria encompassed duplicate articles, letters, case reports, reviews, meta-analyses, and irrelevant titles or abstracts. Additionally, studies with incomplete outcome data that hindered accurate assessment were excluded. Two researchers independently assessed article titles and abstracts using predefined inclusion and exclusion criteria.

### Quality assessment

Using the Cochrane Risk of Bias Tool for randomized trials (20), a pair of independent researchers assessed the quality levels of the selected studies. For nonrandomized trials, two reviewers independently assessed quality using the Newcastle-Ottawa Scale (NOS) (21). If any discrepancies arose, a third researcher was consulted to resolve the issue.

### Data extraction

Two independent researchers meticulously extracted data from each included study, focusing on the following elements: author, publication year, country of origin, study design, outcomes, type of steroid used, follow-up duration, comparison group, mean age, gender distribution, and the number of participants. Any disagreements between the researchers were resolved through thorough discussions, ultimately achieving consensus.

### Outcome measures

The primary outcome measure in this study was the vertigo control rates. This was assessed according to the criteria defined by the American Academy of Otolaryngology-Head and Neck Surgery (AAO-HNS) in 1995 (22). Specifically, we prioritized using Class A as the indicator for vertigo control. If Class A data were not available, we defaulted to combining Class A and Class B to determine the vertigo control rate. Additionally, we evaluated the vertigo control rates at 6 months and 12 months post-treatment. Another evaluation outcome was the change in the PTA before and after treatment. PTA is a measure used to assess auditory function, expressed in decibels (dB); higher values indicate reduced auditory sensitivity and more severe hearing loss.

### Statistical analysis

For continuous outcomes, we determined pooled effect estimates by calculating the weighted mean difference (WMD), and for binary outcomes, we used the risk ratio (RR), each accompanied by their respective 95% confidence intervals (CIs). To evaluate heterogeneity within and between groups, we employed the Cochrane *Q* and *I*^2^ statistics (23). To assess heterogeneity both within and between groups, we applied the Cochrane *Q* and *I*^2^ statistics. If studies exhibit significant heterogeneity (*I*^2^ ≥ 50%), a random-effects model would be utilized (24, 25). Furthermore, meta-regression analysis and leave-one-out sensitivity analysis were conducted to determine the sources of heterogeneity (26). Conversely, in cases of low heterogeneity (*I*^2^ < 50%), a fixed-effects meta-analysis would be conducted for comparison (27).

Publication bias was evaluated using a funnel plot and Egger’s test (28). For all statistical tests, a *p*-value below 0.05 was deemed statistically significant. Statistical analyses were performed using the R version 4.3.1.

## Results

### Literature search and study selection

**Figure 1:**
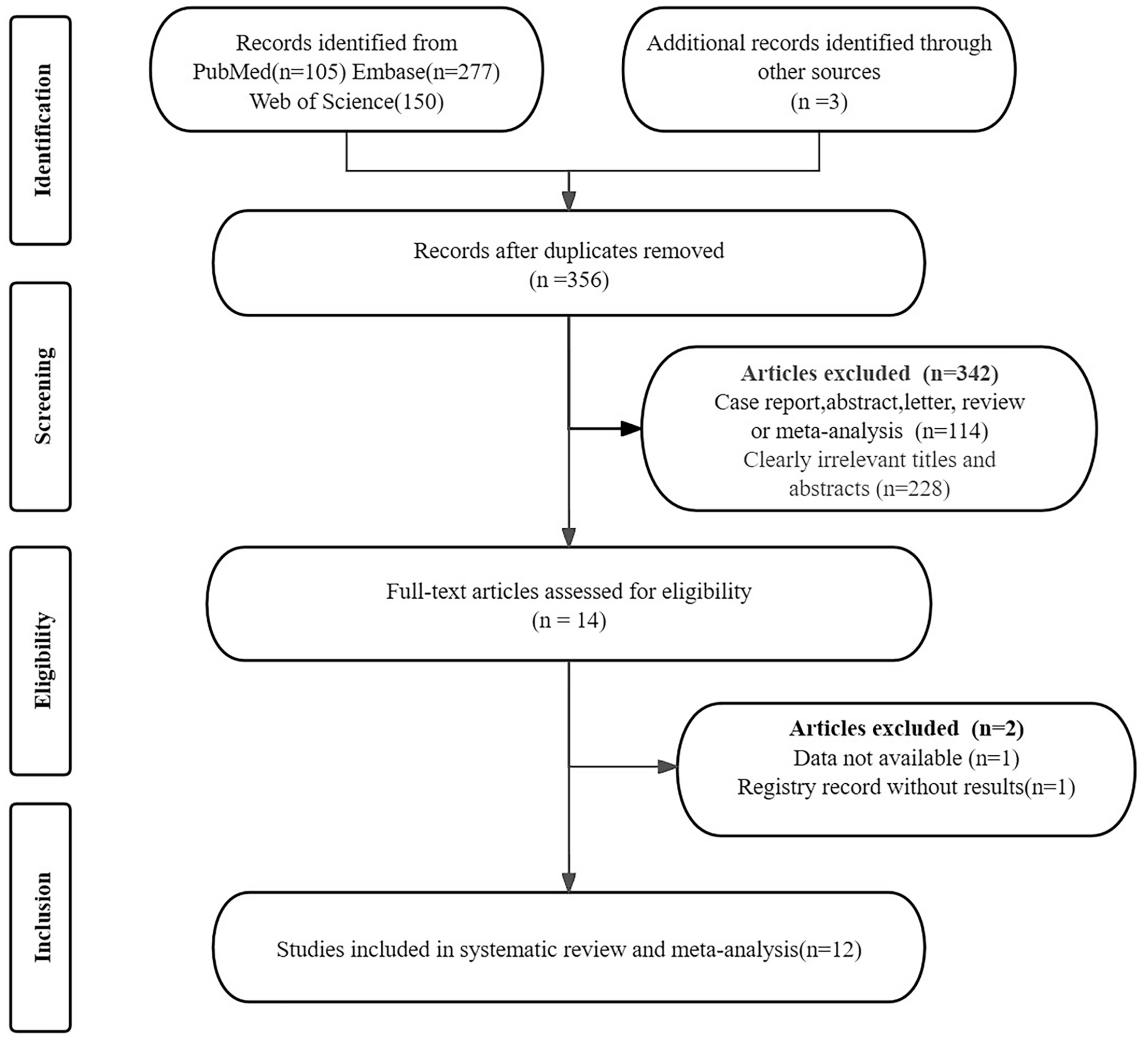
PRISMA flow diagram illustrating the study selection process. PRISMA, preferred reporting items for systematic reviews and meta-analyses.

The initial search yielded 535 publications. After removing 179 duplicates and excluding 342 ineligible studies, 14 articles remained. Upon further review of these full texts, 2 additional studies were excluded: one due to unavailable data and one due to a registry record lacking result. This process resulted in the selection of 12 studies for the analysis of the efficacy in Meniere’s disease (11, 12, 15, 16, 29–36). Figure 1 presents the PRISMA flow diagram, which illustrates the selection process.

### Study description and quality assessment

In these eligible studies on Meniere’s disease, we evaluated data from 5 randomized controlled trials, 2 retrospective studies, and 5 prospective studies, encompassing a total of 694 patients. Seven studies were conducted in Asian countries and five in non-Asian countries. The number of patients in each study ranged from 20 to 124, with study durations varying from 3 months to 24 months, and the average age of participants ranging from 36 to 54.3 years. All studies involved interventions with gentamicin and corticosteroids, specifically dexamethasone and methylprednisolone. The efficacy measures in all studies included either vertigo control rate or pure tone average. Table 1 succinctly summarizes their characteristics.

**Table 1:** The study characteristics of the included studies.

Author	Year	Country	Study design	Outcome	Steroid type	Follow-up time	Comparison	Mean age ± SD or Mean age (range)	Male/Female	Number of patients
Manimaran et al.	2020	India	Retrospective	(1)(2)	Dexamethasone	12 months	Gentamycin	46.45 ± 11.41	14/17	13
							Steroid			18
Thomas et al.	2022	India	RCT	(1)	Methylprednisolone	3 months	Gentamycin	44.36 ± 10.7	7/4	11
							Steroid	42.27 ± 7.7	6/5	11
Naples et al.	2018	USA	Retrospective	(1)(2)	Dexamethasone	>6 months	Gentamycin	NA	43/18	70
							Steroid	NA	15/18	33
Sennaroglu et al.	2001	Turkey	Prospective	(1)	Dexamethasone	18 months	Gentamycin	42 (30–61)	8/8	16
							Steroid	36 (28–72)	16/8	24
Casani et al.	2012	Italy	RCT	(1)(2)	Dexamethasone	24 months	Gentamycin	54.2 ± 12.9	11/21	33
							Steroid	53.7 ± 12.9	10/18	28
Guo et al.	2016	China	Prospective	(1)(2)	Dexamethasone	24 months	Gentamycin	43.6 ± 0.9	26/34	60
							Steroid	41.3 ± 0.7	34/32	66
Gabra et al.	2013	Canada	Prospective	(1)(2)	Methylprednisolone	12 months	Gentamycin	54.3	19/28	47
							Steroid	53	11/31	42
Wang et al.	2017	China	Prospective	(1)(2)	Dexamethasone	24 months	Gentamycin	51.5 ± 10.9	12/23	35
							Steroid	50.6 ± 11.2	14/21	35
Akkuzu et al.	2006	Turkey	Prospective	(1)	Dexamethasone	NA	Gentamycin	46	11/13	24
							Steroid	50.4	8/13	21
ElBeltagy et al.	2012	Egypt	RCT	(1)(2)	Dexamethasone	12 months	Gentamycin	42 (29–57)	19/11	15
							Steroid			15
Patel et al.	2016	UK	RCT	(1)(2)	Methylprednisolone	6 months	Gentamycin	53.3 ± 10.8	15/15	30
							Steroid	51.6 ± 10.2	20/10	30
Sarafraz et al.	2015	Iran	RCT	(1)(2)	Methylprednisolone	3 months	Gentamycin	51.10 ± 13.98	5/5	10
							Steroid	46.10 ± 7.98	3/7	10

NA, not available; RCT, randomized controlled trial; (1) rate of vertigo control; (2) pure tone average change.

**Figure 2:**
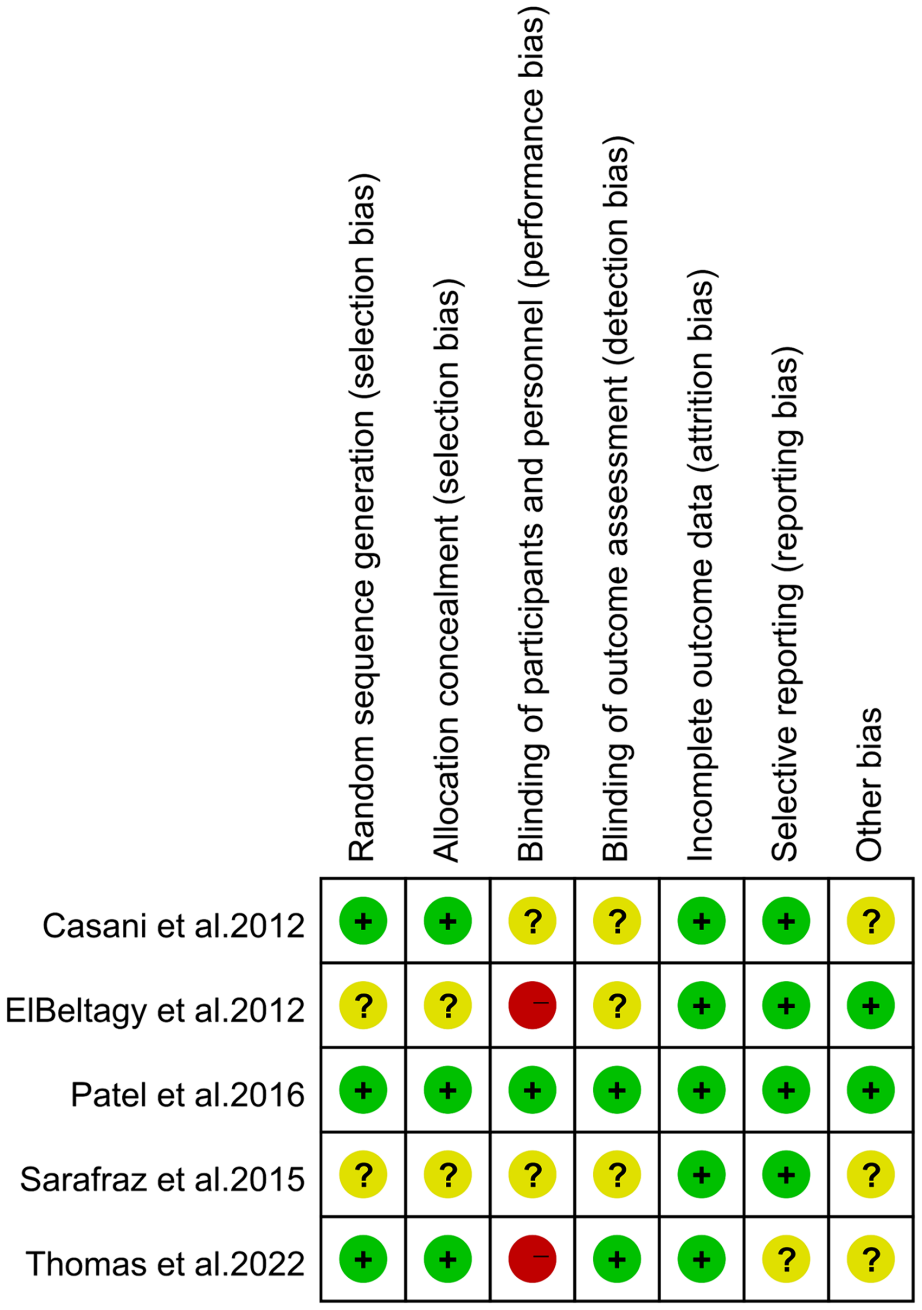
Risk of bias of the included studies using the Cochrane risk of bias tool for randomized trials.

Figure 2 and Supplementary Table S2 display the risk of bias in each study, evaluated using the Cochrane Risk of Bias Tool and the Newcastle-Ottawa Scale (NOS). The assessment indicates that high risk is predominantly associated with the blinding of participants and personnel, also known as performance bias. This high risk arises because blinding was not implemented for the study subjects and the individuals administering the interventions in two of the studies (12, 35).

For the NOS assessment, all articles received a total score of 9 points. Overall, the included studies demonstrated an acceptable level of quality.

### Quantitative analysis of vertigo control rates

**Figure 3:**
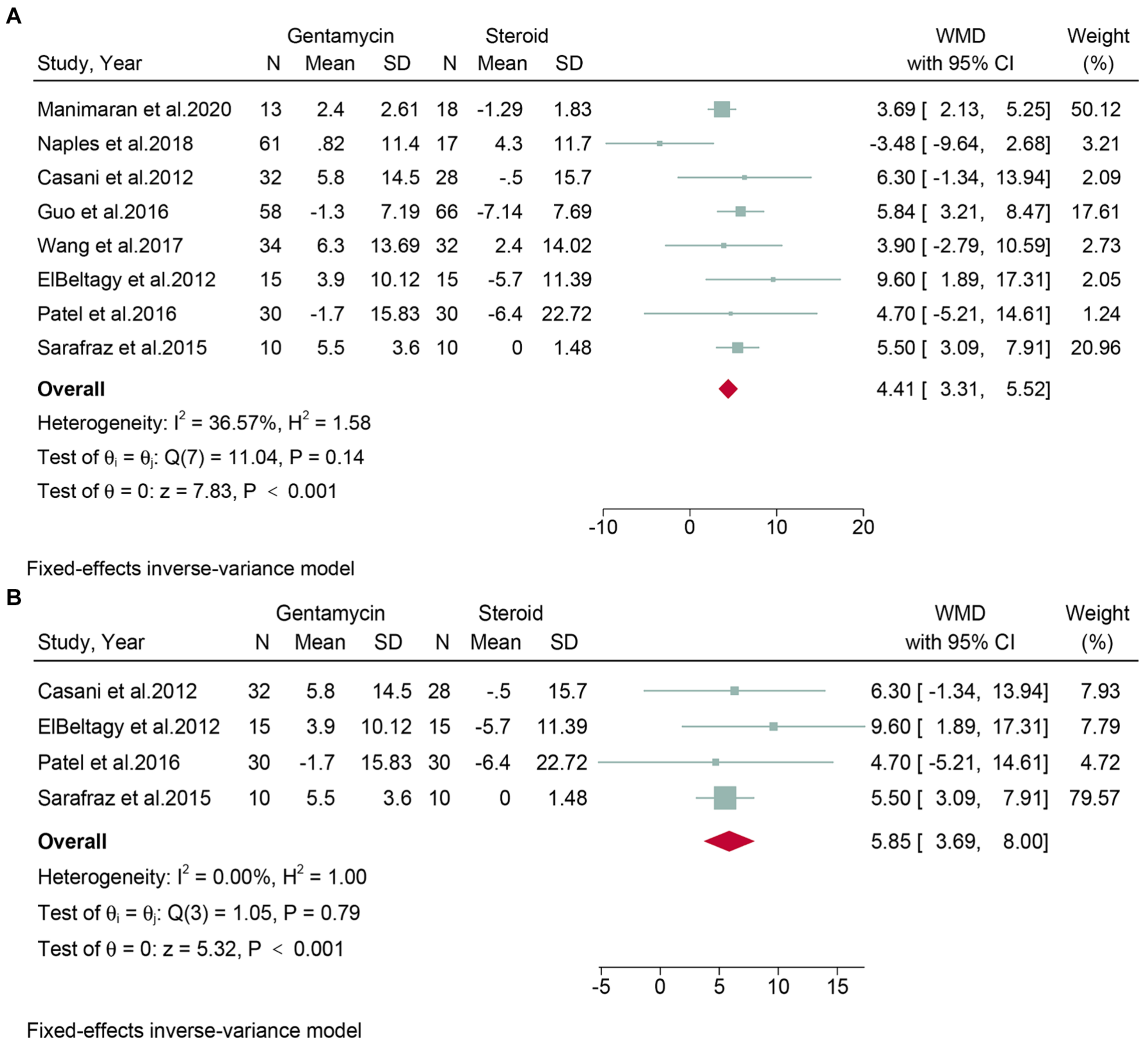
Forest plot of vertigo control rates in patients with Meniere’s disease treated with intratympanic gentamicin and intratympanic corticosteroids. **(A)** All studies and **(B)** randomized controlled trials.

Twelve studies, encompassing a total of 694 patients, assessed the rates of vertigo control. Due to the high heterogeneity observed (*I*^2^ = 55.00%, *p* = 0.01), a random-effects model was employed. The meta-analysis revealed that gentamicin group have significantly higher vertigo control rates (RR: 1.36, 95% CI: 1.13 to 1.65, *p* < 0.001) compared to corticosteroid group (Figure 3A). The leave-one-out sensitivity analysis shown that the result was stable after omitting studies one-by-one (Supplementary Figure S1). Meta-regression analysis revealed that vertigo control rates were not significantly influenced by any of the four covariates: number of patients included, region, study design, steroid type, and follow-up time (Table 2). Based on the statistical analysis using Egger’s test (*p* = 0.02) and the visual representation on the funnel plot, there was evidence of slight publication bias (Supplementary Figure S2).

**Table 2:** Subgroup analysis and meta-regression analysis.

Covariate	Studies, *n*	Vertigo complete control rates RR (95%CI)	Meta-regression *p* value
Number of patients included			0.86
>50	6	1.36 (1.04–1.77)	
≤50	6	1.37 (1.02–1.86)	
Region			0.50
Asia	7	1.29 (0.98–1.69)	
Non-Asia	5	1.47 (1.11–1.95)	
Study design			0.32
Retrospective	2	1.18 (0.85–1.64)	
Prospective	5	1.36 (1.01–1.84)	
Randomized controlled trial	5	1.62 (1.06–2.47)	
Steroid type			0.84
Dexamethasone	8	1.35 (1.07–1.70)	
Methylprednisolone	4	1.44 (0.94–2.21)	
Follow-up time			0.79
≥12 months	8	1.40 (1.10–1.77)	
<12 months	3	1.38 (0.75–2.53)	

Subgroup analyses were conducted on randomized controlled trials. Five studies, encompassing 192 patients, evaluated vertigo control rates. Due to high heterogeneity (*I*^2^ = 56.86%, *p* = 0.04), a random-effects model was used. The meta-analysis indicated that the gentamicin group demonstrated better vertigo control compared to the corticosteroid group (RR: 1.62, 95% CI: 1.06 to 2.47, *p* = 0.03) (Figure 3B).

### Quantitative analysis of vertigo control rates at 6 months and 12 months post-treatment

**Figure 4:**
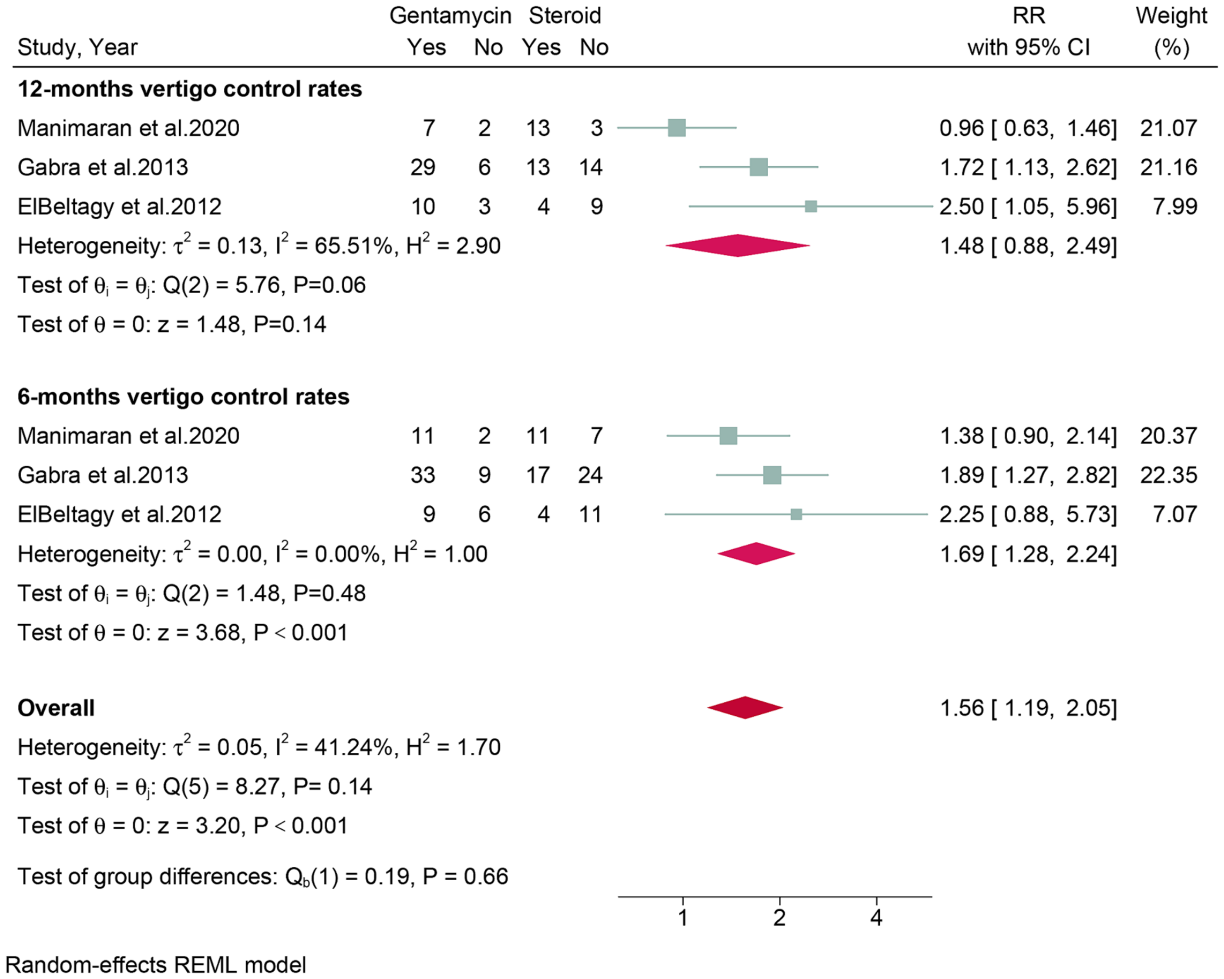
Forest plot of vertigo control rates in patients with Meniere’s disease treated with intratympanic gentamicin and intratympanic corticosteroids in six and twelve mouths.

Three studies, comprising a total of 150 patients, assessed vertigo control rates at 6 months post-treatment. The meta-analysis revealed that the gentamicin group had superior vertigo control rates at 6 months post-treatment (RR: 1.69, 95% CI: 1.28 to 2.24, *p* < 0.001) compared to the corticosteroid group (Figure 4).

Three studies, encompassing a total of 150 patients, evaluated vertigo control rates at 12 months post-treatment. The meta-analysis showed no significant difference (RR: 1.48, 95% CI: 0.88 to 2.49, *p* = 0.14) in vertigo control rates between the gentamicin group and the corticosteroid group after 12 months of treatment (Figure 4).

### Quantitative analysis of the change in pure tone average

**Figure 5:**
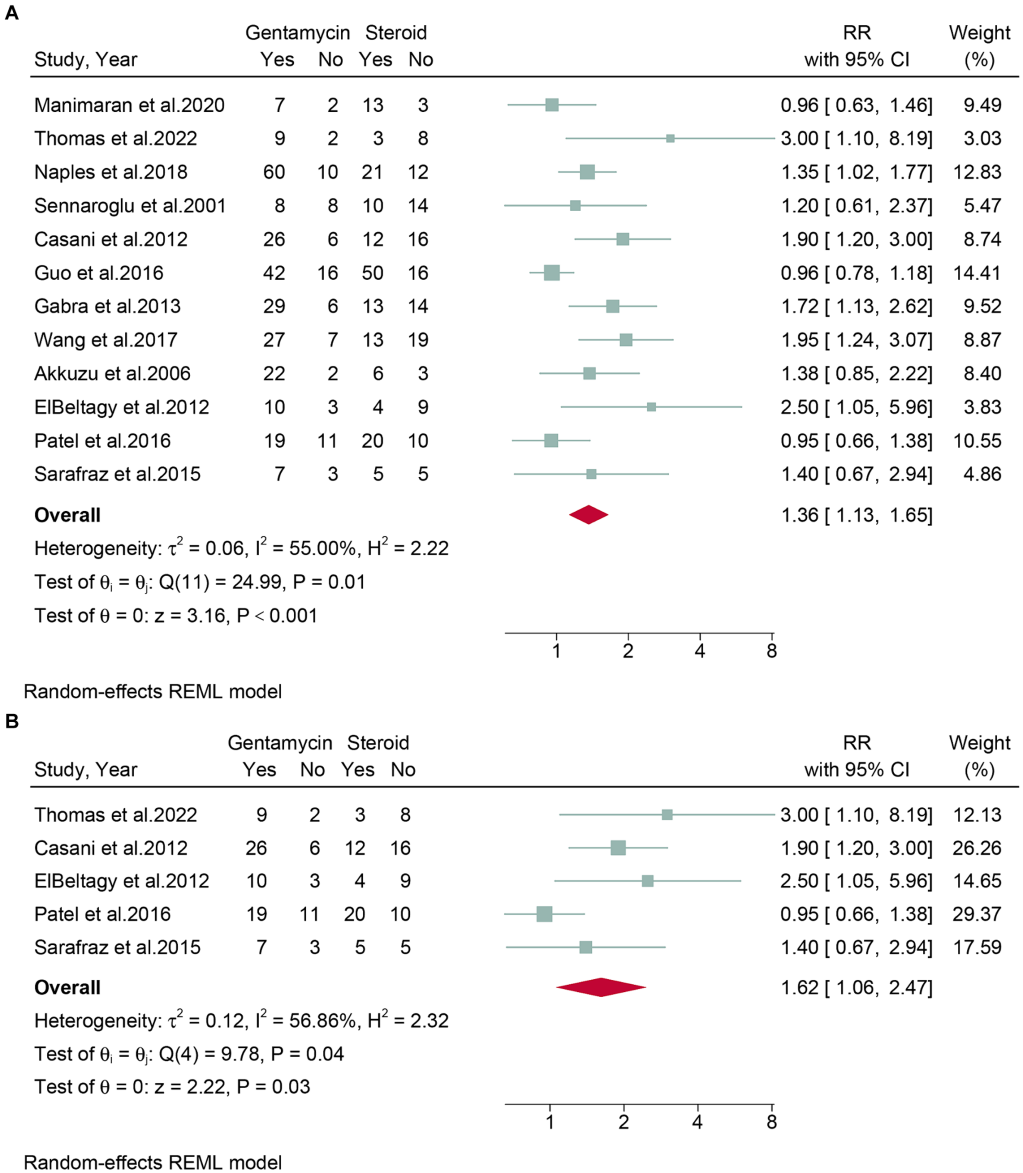
Forest plot of changes in pure tone average in patients with Meniere’s disease treated with intratympanic gentamicin and intratympanic corticosteroids. **(A)** All studies and **(B)** randomized controlled trials.

Eight studies, encompassing a total of 498 patients, assessed the change in PTA. Given the low heterogeneity observed (*I*^2^ = 36.57%, *p* = 0.14), a fixed-effect model was applied. The meta-analysis showed that the corticosteroid group had a superior protective effect on hearing compared to the gentamicin group (WMD: 4.41, 95% CI: 3.31 to 5.52, *p* < 0.001) (Figure 5A). The leave-one-out sensitivity analysis shown that the result was stable after omitting studies one-by-one (Supplementary Figure S3). No detectable publication bias was found, as evidenced by the funnel plot and Egger’s test (*p* = 0.88) (Supplementary Figure S4).

Subgroup analyses were conducted on randomized controlled trials. Four studies, encompassing 170 patients, evaluated the change in PTA. Due to low heterogeneity (*I*^2^ = 0.00%, *p* = 0.79), a fixed-effects model was used. The meta-analysis concluded that the corticosteroid group was superior to the gentamicin group in terms of hearing protection (WMD: 5.85, 95% CI: 3.69 to 8.00, *p* < 0.001) (Figure 5B).

## Discussion

The 2020 Clinical Practice Guidelines for Meniere’s Disease by the American Academy of Otolaryngology-Head and Neck Surgery (AAO-HNS) state that the level of complete vertigo control (class A) was lower for intratympanic steroid therapy (31–90% of subjects) compared to intratympanic gentamicin therapy (70–87% of subjects) (6). However, the Chinese Clinical Practice Guideline for Intratympanic Medication indicates that both treatments effectively reduce vertigo rates, without significant differences (37). It also highlights that steroids provide superior hearing protection, recommending gentamicin only for cases unresponsive to steroids. In recent years, some studies have suggested that gentamicin has superior vertigo control rates compared to steroids (12, 15), while others report equivalent efficacy between the two treatments (30, 32). Consequently, there is an ongoing debate about their relative effectiveness in managing Meniere’s disease.

The meta-analysis revealed that the intratympanic gentamicin group had significantly higher vertigo control rates (RR: 1.36, *p* < 0.001) compared to the intratympanic corticosteroid group. The superior vertigo control seen with gentamicin is likely due to its selective vestibulotoxicity, where it damages vestibular hair cells, thus reducing the abnormal signals contributing to vertigo—a process known as “chemical ablation.” However, this effectiveness comes with a risk of hearing loss, particularly at high cumulative doses and short intervals between injections, due to its potential cochleotoxic effects. Conversely, the analysis indicated that the corticosteroid group had a superior protective effect on hearing (WMD: 4.41, *p* < 0.001) compared to the gentamicin group. IT corticosteroids offer hearing preservation through their anti-inflammatory and immunomodulatory properties, which reduce inner ear inflammation, pressure, and fluid buildup, thus protecting the auditory structures. This protective role is likely due to corticosteroids’ ability to decrease the release of inflammatory mediators and vascular permeability, maintaining cochlear homeostasis.

Our meta-analysis expands upon previous evaluations by Jiang et al. (17), Ahmadzai et al. (8), and Hao et al. (18) by incorporating a broader range of studies. Jiang et al. (17) demonstrated the superiority of intratympanic gentamicin over corticosteroids in vertigo control (OR 3.08, 95% CI: 2.05–3.65, *p* < 0.01), aligning with our findings. However, their inconsistent results regarding hearing improvement highlight limitations in data stability, with their sensitivity analysis showing instability through stepwise exclusion. Our study, with a more extensive dataset, consistently shows corticosteroids superior in hearing preservation, with lower heterogeneity and no publication bias. Another meta-analysis conducted by Ahmadzai et al. (8) that evaluated the efficacy of various treatments for Meniere’s disease, restricting their analysis to randomized controlled trials. In their comparison of gentamicin and corticosteroids, they included only three studies. In contrast, our study incorporates a broader range of study designs, including prospective, retrospective, and RCT studies. We conducted comprehensive efficacy evaluations for vertigo control and hearing preservation across all study types, as well as separate analyses specifically for RCTs. Our results, whether overall or specifically for RCTs, were stable.

In 2021, Hao et al.’s network meta-analysis (18) found no significant difference in vertigo control between treatments (RR, 1.21; 95% CI 0.92–1.58; *p* = 0.17), which contrasts with our meta-analysis revealing a significant advantage of intratympanic gentamicin over corticosteroids in vertigo control for Meniere’s disease. This discrepancy may be attributable to their inclusion of only four studies (170 patients), potentially limiting the robustness of their results. Furthermore, their criteria for hearing control, based on AAO-HNS 1995 criteria, considered both Class A and B as effective vertigo control, whereas our study prioritized Class A, which may have led to differing outcomes. Our findings suggest that gentamicin may offer superior effectiveness in achieving complete vertigo control (Class A) compared to corticosteroids. Further studies in this issue are needed.

Both treatments are relatively accessible, though intratympanic gentamicin may be slightly more available due to its longer history of use in otology (38). Intratympanic corticosteroid is generally considered safer, particularly concerning ototoxicity, which is a significant risk with intratympanic gentamicin (18). Our results further support this conclusion. The potential for gentamicin-induced hearing loss necessitates careful patient selection and monitoring (39). Our findings suggest that intratympanic gentamicin has a superior effect on vertigo control compared to intratympanic corticosteroid. However, it should be noted that the high heterogeneity in vertigo control outcomes indicates a need for further research to achieve more robust conclusions. Due to the ototoxic nature of gentamicin, its use in patients should be approached with caution, despite its higher efficiency. The choice of specific treatment methods should be based on the individual patient’s situation.

Our meta-analysis presents several limitations that must be acknowledge. First, despite our efforts to identify sources of heterogeneity in vertigo control rates through meta-regression, no significant impact was observed from variables such as the number of patients included, region, study design, steroid type, or follow-up time. This suggests that other factors may contribute to the heterogeneity in treatment outcomes. Second, the limited number of available studies necessitated the inclusion of some retrospective and prospective studies in our analysis. To address this, we conducted a subgroup analysis focusing solely on RCTs, which yielded robust results compared to the overall results. Future research should prioritize larger sample size RCTs to provide more definitive conclusions.

## Conclusion

Our study suggests that the intratympanic gentamicin group achieves higher vertigo control rates, whereas the corticosteroid group demonstrates better improvement in pure tone averages. However, the high heterogeneity in vertigo control rates warrants caution. Larger sample-sized randomized controlled trials are needed to further validate these findings.

## Data Availability

The original contributions presented in the study are included in the article/Supplementary material, further inquiries can be directed to the corresponding author.
