# Supplementary material for: Comparative efficacy of intratympanic gentamicin and intratympanic corticosteroid in the treatment of Meniere’s disease: a systematic review and meta-analysis
Source: Front Neurol. 2024 Sep 12;15:1471010. doi: 10.3389/fneur.2024.1471010 (PMC11424416; doi:10.3389/fneur.2024.1471010)

Supplementary Table 1 Search strategy in PubMed, Embase, and Web of Science

| Database | Search strategy |
| --- | --- |
| PubMed  (105) | (“Gentamicins”[Mesh] OR “Gentamicin”[Title/Abstract] OR “Gentamycin”[Title/Abstract] OR “Gentacycol”[Title/Abstract] OR “Genticin”[Title/Abstract] OR “Gentamycin”[Title/Abstract]) AND ("Steroids"[Mesh] OR "Glucocorticoids"[Mesh] OR "Methylprednisolone"[Mesh] OR “Adrenal Cortex Hormones”[Mesh] OR “Dexamethasone” [Title/Abstract] OR “steroids”[Title/Abstract] OR “corticosteroids”[Title/Abstract] OR “glucocorticoid”[Title/Abstract] OR “dexamethasone” [Title/Abstract] OR “methylprednisolone”[Title/Abstract]) AND ("Meniere Disease"[Mesh] OR “Meniere's Disease”[Title/Abstract] OR “meniere disease”[Title/Abstract]) |
| Embase  (277) | (‘gentamicin’/exp OR ‘Gentamicin’:ab,ti OR ‘Gentamycin’:ab,ti OR ‘Gentacycol’:ab,ti OR ‘Genticin’:ab,ti OR ‘Gentamycin’:ab,ti) AND (‘steroid’/exp OR ‘corticosteroid’/exp OR ‘glucocorticoid’/exp OR ‘dexamethasone’/exp OR ‘methylprednisolone’/exp OR ‘steroids’:ab,ti OR ‘corticosteroids’:ab,ti OR ‘dexamethasone’:ab,ti OR ‘methylprednisolone’:ab,ti) AND (‘Meniere disease’/exp OR ‘Meniere’:ab,ti OR ‘meniere disease’:ab,ti) |
| Web of Science  (150) | ((TS=(“Gentamicins” OR “Gentamicin” OR “Gentamycin” OR “Gentacycol” OR “Genticin” OR “Gentamycin”)) AND TS=("Steroids" OR "Glucocorticoids" OR "Methylprednisolone" OR “Adrenal Cortex Hormones” OR “Dexamethasone” OR “steroids”] OR “corticosteroids” OR “glucocorticoid” OR “dexamethasone” OR “methylprednisolone”)) AND TS=("Meniere Disease" OR “Meniere's Disease” OR “meniere disease”) |

| Study, year | Study type | Representativeness  of the exposed cohort | Selection of the nonexposed  cohort | Ascertainment  of exposure | Outcome of interest was not present at  start of study | Comparability of cohorts on the basis of  the design or analysis | Assessment of outcome | Was follow-up long  enough for outcomes to occur | Adequacy of follow-up of cohorts | Total  score |
| --- | --- | --- | --- | --- | --- | --- | --- | --- | --- | --- |
| Manimaran et al. 2020 | Retro | 1 | 1 | 1 | 1 | 2 | 1 | 1 | 1 | 9 |
| Naples et al. 2018 | Retro | 1 | 1 | 1 | 1 | 2 | 1 | 1 | 1 | 9 |
| Sennaroglu et al. 2001 | Pro | 1 | 1 | 1 | 1 | 2 | 1 | 1 | 1 | 9 |
| Guo et al. 2016 | Pro | 1 | 1 | 1 | 1 | 2 | 1 | 1 | 1 | 9 |
| Gabra et al. 2013 | Pro | 1 | 1 | 1 | 1 | 2 | 1 | 1 | 1 | 9 |
| Wang et al. 2017 | Pro | 1 | 1 | 1 | 1 | 2 | 1 | 1 | 1 | 9 |
| Akkuzu et al. 2006 | Pro | 1 | 1 | 1 | 1 | 2 | 1 | 1 | 1 | 9 |

Supplementary Table 2 NOS quality assessment results of the nonrandomized studies.

Supplementary Figure 1 Leave-one-out sensitivity analysis plot of vertigo control rates in patients with Meniere's Disease treated with intratympanic gentamicin and intratympanic corticosteroids.


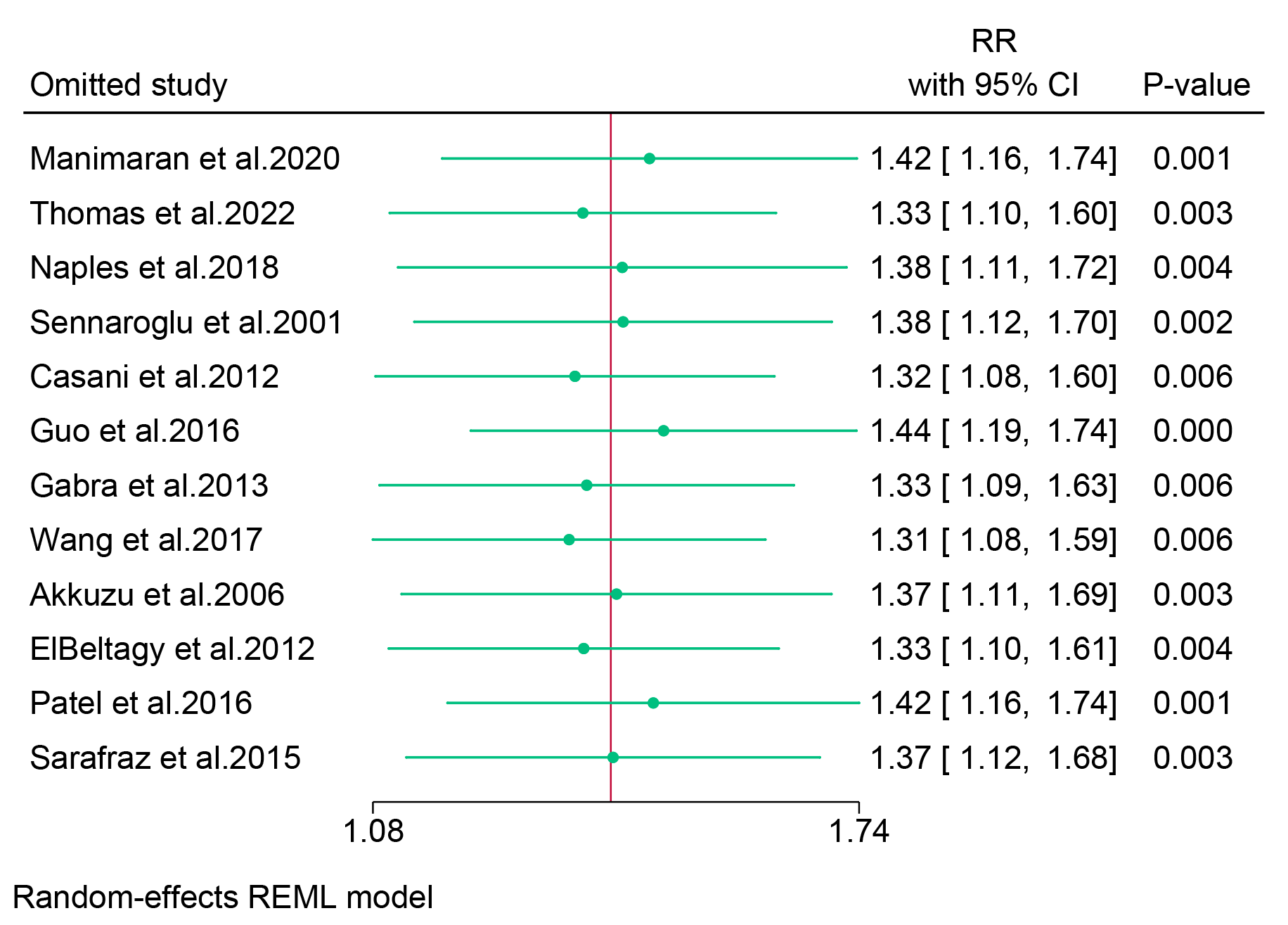


Supplementary Figure 2 Funnel plot of vertigo control rates in patients with Meniere's Disease treated with intratympanic gentamicin and intratympanic corticosteroids.


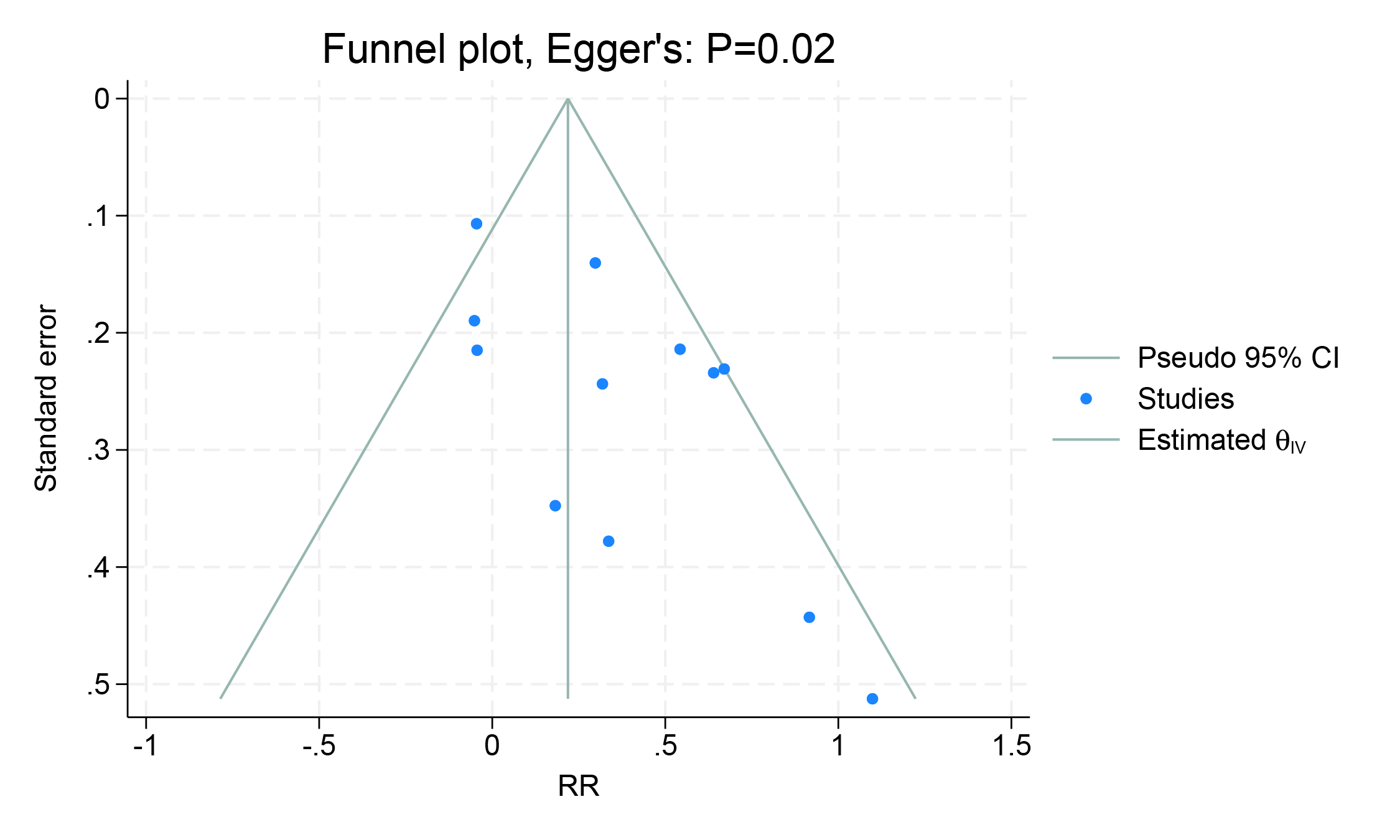


Supplementary Figure 3 Leave-one-out sensitivity analysis plot of changes in pure tone average in patients with Meniere's Disease treated with intratympanic gentamicin and intratympanic corticosteroids.


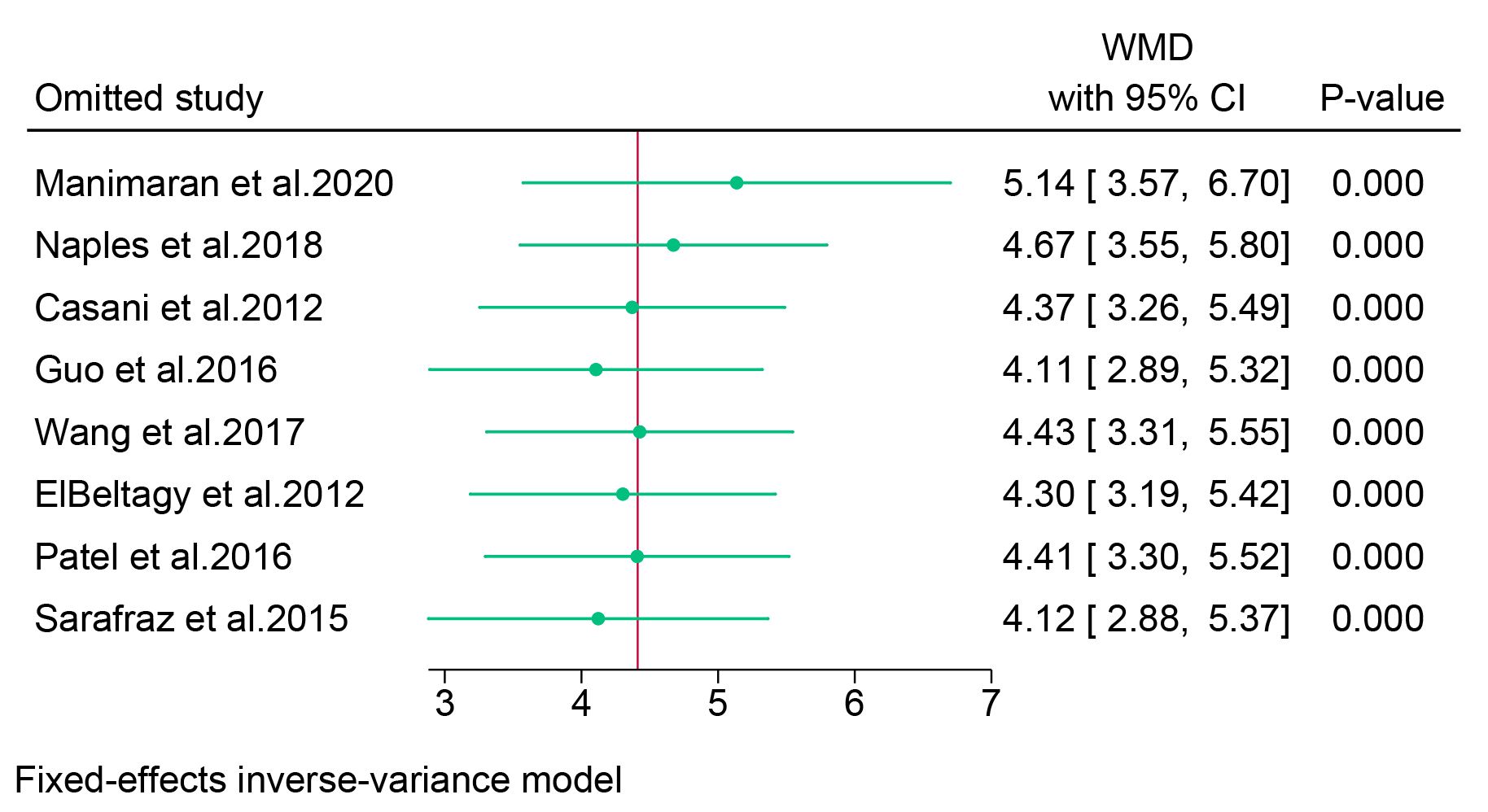


Supplementary Figure 4 Funnel plot of changes in pure tone average in patients with Meniere's Disease treated with intratympanic gentamicin and intratympanic corticosteroids.


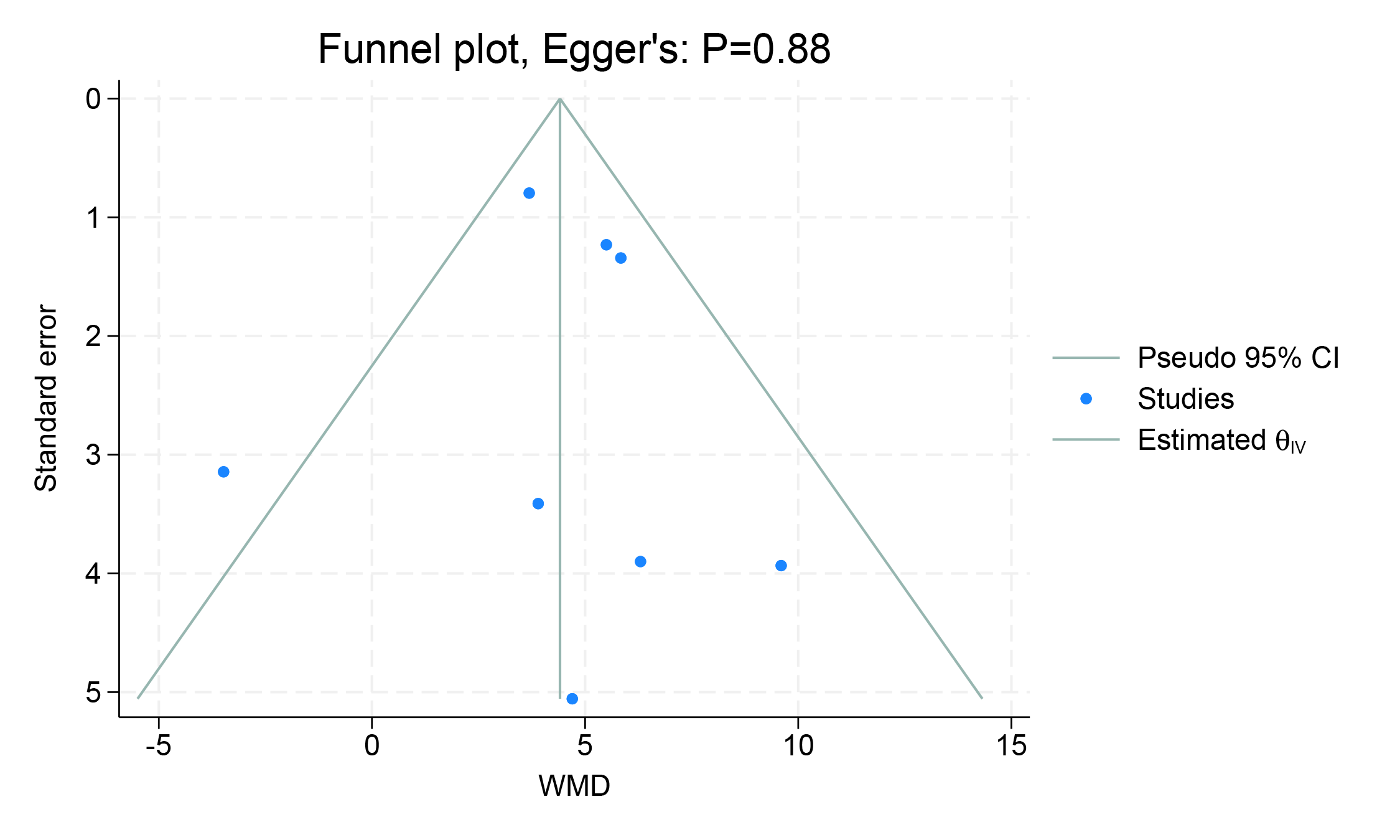

Supplement: Supplementary file 1 [file Data_Sheet_1.docx]
